# Left atrial strain for predicting recurrence in patients with non-valvular atrial fibrillation after catheter ablation: a single-center two-dimensional speckle tracking retrospective study

**DOI:** 10.1186/s12872-022-02916-y

**Published:** 2022-11-05

**Authors:** Yuanzhi Li, Yidan Li, Lanlan Sun, Xiaoguang Ye, Qizhe Cai, Weiwei Zhu, Dichen Guo, Xueyan Ding, Jiangtao Wang, Xiuzhang Lv

**Affiliations:** 1 Department of Echocardiography, Beijing Chao Yang Hospital, Capital Medical University, No. 8 Gongren Tiyuchang Nanlu, Chao Yang District, Beijing, 100020 China; 2 General Electric Medical System (China), Clinical Education Team, Beijing, 100176 China

**Keywords:** Atrial fibrillation, Catheter ablation, Speckle tracking cardiography, Recurrence

## Abstract

**Background:**

Although catheter ablation (CA) is an effective treatment for non-valvular atrial fibrillation (AF), a good many of patients still have a recurrence following post-operation. Prediction of AF recurrence by evaluating left atrial (LA) phase function with speckle tracking echocardiography (STE) may be helpful for risk stratification and clinical management for AF patients. Therefore, the current study aimed to assess the prognostic value of LA strains in non-valvular AF patients after CA.

**Methods:**

A total of 95 non-valvular AF patients (70.5% paroxysmal AF, 56.8% males, mean age 63.2 ± 9.7 years) were included in this retrospective study between October 2019 and August 2020. Transthoracic echocardiography was performed in all the subjects and STE was used to analyze the LA reservoir strain (LASr), LA conduit strain (LAScd) and LA contractile strain (LASct) during different phases before CA. Patients were followed up with until January 2022. The endpoint was AF recurrence.

**Results:**

Over a median follow-up period of 26.0 months (interquartile range, 24.7–26.7 months), 26 patients experienced recurrence and 69 stayed in sinus rhythm. Compared with no-recurrence group, maximum volume of LA (LAVmax), minimum volume of LA (LAVmin) and LA volume index (LAVI) were increased in the recurrence group, while LAEF, LASr and LASct were worsened (*P* < 0.05). Multivariable logistic regression analysis revealed that LASct was an independent predictor of AF recurrence (odds ratio, 0.89; 95% confidence interval (CI), 0.82–0.97; *P* = 0.007) and receiver operating characteristic (ROC) curve analysis showed an area under the curve of LASct<8% was 0.70 (95% CI, 0.59–0.79; *P* = 0.0008).

**Conclusions:**

LASct was of independent predictive value of AF recurrence. LA function assessed by STE may contribute to the risk stratification for AF patients and selection of suitable patients for CA.

## Introduction

Atrial fibrillation (AF) is a common arrhythmia with the morbidity and mortality increasing in the wake of the aged population all over the world that bring about poor prognosis and heavy healthcare burden. Great prevalence of AF in the general population, especially in the elderly [1]. Change of atrial myocardium both in structure and function may lead to disordered intra-atrial conduction and electrical atrial remodeling, resulting the increasement of susceptibility to AF and need for different interventions [2, 3]. It has established that AF exerts the damage on left atrial (LA) structure and function, leading to enlarged LA volume and LA dysfunction [4, 5]. LA and left ventricular (LV) remodeling has already emerged in paroxysmal AF stage [4]. Fibrosis promotes atrial remodeling, increasing the atrial re-entrant circuits and AF burden, raising the recurrence of AF after operation [6, 7]. Some studies have proved prognostic value of LA function for the prediction of incident AF and postoperative recurrence [8, 9].

Various treatments for AF are aim to prevent or decrease the thromboembolism, reduce heart rate and control rhythm. At present, catheter ablation (CA) is more effective in controlling AF and reducing recurrence according to the European Society of Cardiology guidelines recommend [1]. LA, a vital element of the arterial-ventricular-atrial unit, plays an important role in regulating left ventricular filling by its different phase functions, including reservoir, conduit and booster pump [10]. Echocardiography is real-time, reproducible and availably deemed to the noninvasive method for LA functional evaluation in the diagnosis and management of AF. Speckle tracking cardiography (STE) provides a novel way to quantify the LA myocardial strain and assess each phase function separately. Because some patients still recur to AF rhythm after a successful CA, identifying patients at risk of AF recurrence is of clinical values and may be beneficial to the improvement of risk stratification and individualized management. It is known that enlarged LA diameter was an independent predictor of worse AF ablation outcomes [11]. LA reservoir strain (LASr) and LA conduit strain (LAScd), but not LA contractile strain (LASct), were important indices that derived from STE, demonstrated independent predictors of AF recurrence after therapeutic intervention [9]. However, heterogeneity of measurements for LA strains need a further investigation. The aim of this study was to determine the prognostic value of LA strains that assessed by STE, identify independent predictor and stratify the risks for AF patients.

## Methods

### Study population

Two hundred twenty-one consecutive patients with a diagnosis of AF at Beijing Chaoyang Hospital Heart Center (Beijing, China) between October 2019 and August 2020 were admitted to this research. Ninety-five patients with non-valvular AF who underwent CA and aged>18 years were included finally.

The exclusion criteria meet the following conditions if patients with: 1. valvular AF that involved valve stenosis or experienced a valve replacement surgery; 2. congenital heart disease; 3. cardiomyopathy; 4. implanted pacemaker; 5. poor images resulting unavailable data. With the application of exclusion criteria, a total of 126 patients were initially collected in. Seventeen patients lost follow-up and 5 died from all causes during follow-up. In addition, patients who underwent hybrid minimally invasive surgical and transcatheter ablation (*n* = 5) and atrial appendage closure (*n* = 4) were excluded.

**Fig. 1:**
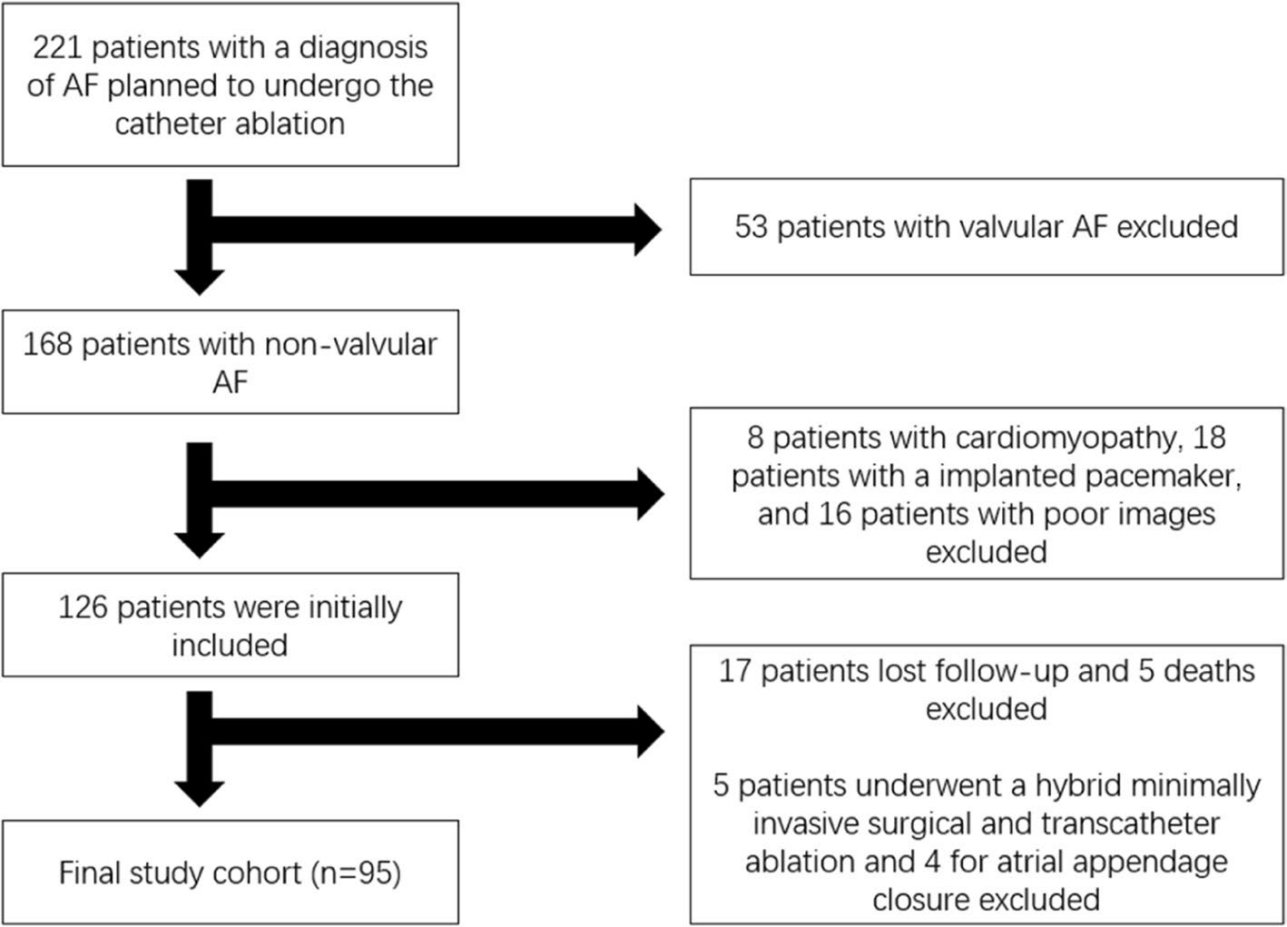
Study protocol

This retrospective study was approved by the ethics committee of Beijing Chao Yang Hospital Affiliated to Capital Medical University. Demographics, basic medications and clinical comorbidities of all patients were collected from the electronic medical record system in Heart Center of Beijing Chao Yang Hospital. Echocardiographic measurements were obtained by offline software. Elaborate characteristics of AF were recorded, including AF types, CHA2DS2-VASc score, HASBLED score, antithrombotic drugs and antiarrhythmic drugs. AF subtype was defined according to ESC current guidelines [1]. If the episodes self-terminating in 7 days was regarded as paroxysmal AF, or lasting longer than 7 days and needing a cardioversion was regarded as persistent AF. The study protocol and participants characteristics are summarized in Fig. 1.

This study of subjects was followed by telephone contact until January 2022 to confirm if had a post-ablation recurrence. The participants were classified into 2 groups: the AF recurrence group and the AF no-recurrence group.

### CA procedures

The CA procedure was performed under general anesthesia. A steerable catheter was inserted in the coronary sinus through the left femoral vein. After transseptal access was secured, an 8.5F sheath (SL1, St. Jude Medical, St Paul, MN, USA) was advanced into the LA and was continuously flushed with saline (20 mL/h) to prevent the formation of thrombi or air embolism, with an activated coagulation time between 250 and 300 seconds during the entire procedure through the administration of intravenous heparin. All patients underwent pulmonary vein angiography, and a real-time three-dimensional electroanatomic LA map was created by the CARTO 3 mapping system (Biosense Webster, Diamond Bar, CA, USA) with the use of a 20-polar mapping catheter (PentaRay™, Biosense Webster, Diamond Bar, CA, USA). Circumferential pulmonary vein isolation (CPVI) of all patients was performed by a contact force-sensing, irrigated 3.5 mm ablation catheter (ThermoCool SmartTouch™, Biosense Webster, Diamond Bar, CA, USA). Radiofrequency energy was delivered from a Stockert generator with a target temperature of 43 °C, and the power limit was set at 30 W to 35 W. If AF continued, the LA sites with the following characteristics were also tagged and ablated after CPVI: (a) continuous (> 5 s), low-amplitude (0.05–0.3 mV), fractionated atrial endocardial electrograms; and (b) more rapid local atrial deflections than those from the adjacent sites. If the recurrent AF unrelated to PV, the LA substrate was ablated during index procedure. The endpoints for the CA procedure were AF terminated and sinus rhythm restored. CPVI was checked by bolus injection of 20 mg adenosine triphosphate in all patients. If a patient received any atrial linear lesions, bidirectional conduction block was confirmed with the use of differential pacing maneuver.

### Echocardiography

#### Conventional data

Transthoracic echocardiography was performed on all patients to evaluate their left cardiac structures and function at the left lateral decubitus position to get standard images by using an E95 ultrasound machine and system (GE Healthcare, Horton, Norway, software version 204). All images were stored in hard disk and conventional parameters were obtained via offline measurement in accordance with the established recommendations [12]. LA and left ventricular (LV) volumetric measurements were processed by the modified Simpson’s method. Left ventricular end-diastolic volume index (LVEDVI) and left ventricular end-systolic volume index (LVESVI) were calculated respectively by the Left ventricular end-diastolic volume (LVEDV) and left ventricular end-systolic volume (LVESV) to body surface area (BSA). Similarly, left atrial volume index (LAVI) was the ratio of the maximum volume of the left atrium (LAVmax) and BSA. Based on tissue Doppler image (TDI) system, E/e’ was estimated by the peak E-wave velocity (E) of mitral valve and average of sum of lateral and septal mitral annular early-diastolic peak velocity (e’) in 4-chamber view.

#### Speckle tracking echocardiography

**Fig. 2:**
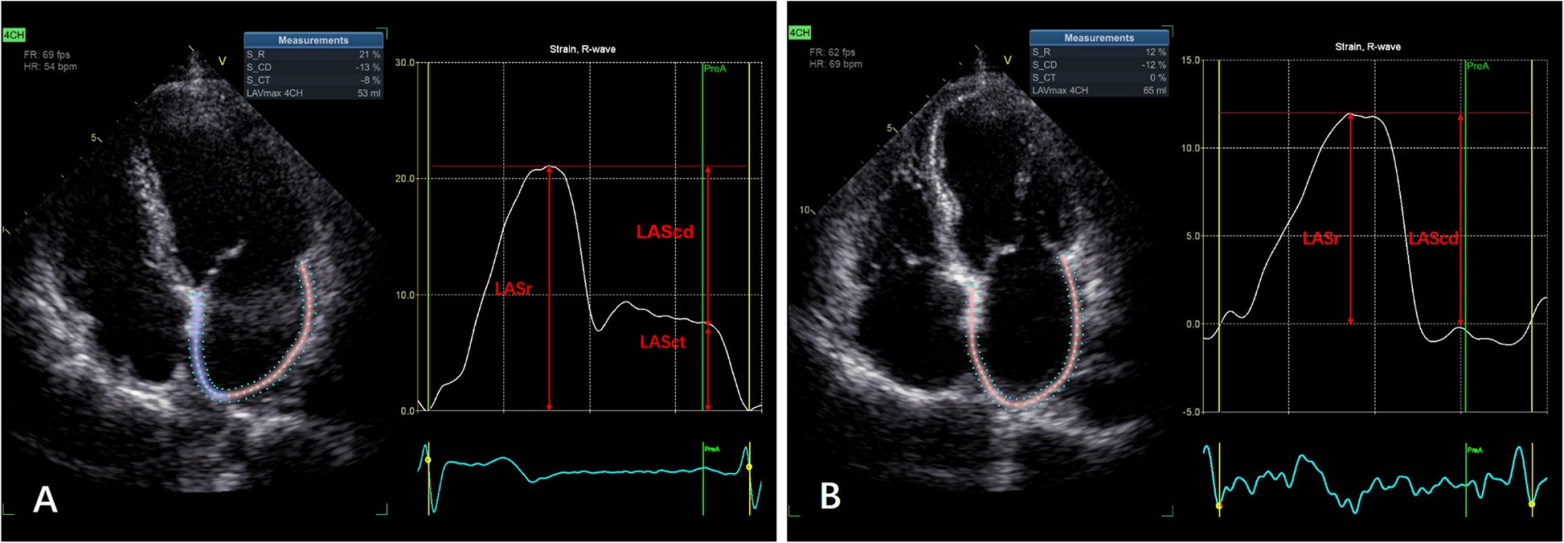
Assessment of LA strain. Panel **A** shows the LA strain curve in an AF patient without recurrence after CA who has LASct. Panel **B** shows the LA strain curve in an AF patient with recurrence after CA who has no LASct

Apical 4-chamber, 2-chamber, and 3-chamber view’s images row data were imported to offline software for analysis. Images were recorded for 6 consecutive cardiac cycles in sinus rhythm and 12 consecutive cardiac cycles for AF patients. The frame rate was at least 60 frames/second. All LA and LV strain measurements were performed by EchoPAC software version 204 (GE Healthcare). The index beat, a simple and practical method, was used to select an optimal heart cycle to evaluate LA and LV dynamics in patients with AF rhythm [13]. Meanwhile, strain offline analyses were performed using the dedicated LA-tracking and LV-tracking software, respectively. When calculating the LV global strain (LVGLS), a region of interest was automatically tracked between LV endocardial and epicardial borders after a click on those 3 apical views sequentially, then a bull eye map along with GLS came out. LA strain was analyzed on apical 4-chamber view recommended by current guidelines with the zero reference at LV end-diastole [14]. Two LA anchor points were dropped at the mitral valve ring and one at the LA roof by a click on 4-chamber view, then the region of interest was appeared and LA strains were automatically generated and the values of strains were shown out, including LA reservoir, conduit as well as contract phase (Fig. 2). If the duration of cardiac cycle between different views was of identifiable difference by system, the strains would not automatically display out neither in LA nor LV strain analysis. Once the region of interest was not matched well with LA or LV wall, a manual modification was needed. In addition, LA volumetric data including LAVmax, minimum volume of left atrium (LAVmin) and left atrial ejection fraction (LAEF) were displayed along with the tracking of the region of LA after a switch to left atrial volume (LAV) layout.

#### Assessment of reproducibility of STE measurements

To evaluate the intra-observer reproducibility of STE data, we randomly drew15 patients from the date sets to make a second analysis after the initial analysis 1 month later. Meanwhile, the data sets of same 15 patients were analyzed by another experienced echocardiographer to assess the inter-observer reproducibility. Inter- and intra-observer variability of LASct was assessed by intraclass correlation coefficients and bias.

#### Follow-up and study outcomes

The follow-up was continued until January 2022 by telephone call and outpatient visits about symptoms and electrocardiograms. The primary outcome was recurrence, which implied that AF rhythm was caught by electrocardiogram or Holter electrocardiogram as well as reappeared symptoms. At the duration of first 3 months, recurrent atrial arrhythmias of AF patients after the catheter ablation were not counted as the primary event.

### Statistical analysis

Continuous variables were displayed as mean ± standard deviation (SD) if normally distributed and as medians with interquartile ranges (Q1–Q3) if not normally distributed. Categorical variables were expressed as frequency and percentages, evaluated by chi-square test. Comparisons between groups were completed with t-test for normally distributed data and with the Mann–Whitney U test for non-normally distributed data in continuous variables. Univariate logistic regression analysis was used to estimate the effect of atrial parameters on the risk of AF recurrence and multivariable regression analysis was used for identifying the independent predictor after the catheter ablation in AF patients. Receiver-operator curves (ROC) was generated from logistic regression to determine the optimal cutoff values of the only independent predictor, and the area under the curve was used to predict recurrence. All tests were calculated from 2-sided, and *P* < 0.05 was considered statistically significant. Statistical analyses were performed by MedCalc 15.0 (MedCalc Software) and SPSS 23.0 (IBM SPSS Statistics, version 23).

## Results

### Study cohort

A total of 95 patients (63.2 ± 9.7 years, 56.8% male, 70.5% paroxysmal AF) were enrolled in this cohort. During a median follow-up period of 26.0 months (interquartile range, 24.7–26.7 months), 26 patients had a recurrence and 69 did not. Table 1 shows the baseline demographics, clinical characteristics and medications of the 2 groups before catheter ablation procedure. At baseline, underlying diseases were existed in AF patients, with 63.2% having hypertension, 18.9% having diabetes mellitus, 73.7% having hyperlipidemia and 45.5% having a history of ischemic disease. Standardized medications were used for AF and its comorbidities, including amiodarone, propafenone, anticoagulation therapies and proper antihypertensive drugs, of insignificant differences between the 2 groups. Further, there were no differences in the subtype of AF, CHA2DS2-VASc score as well as HASBLED score between these groups.

**Table 1:** Demographic and clinical characteristics of the study population

Variables	Total (*N* = 95)	Recurrence (*N* = 26)	No-Recurrence (*N* = 69)	*P* value
Demographics				
Age (years)	63.2 ± 9.7	60.7 ± 8.4	64.2 ± 10.0	0.110
Man, n (%)	54 (56.8%)	15 (57.7%)	39 (56.5%)	0.919
BSA (/m^2^)	1.81 ± 0.17	1.83 ± 0.15	1.81 ± 0.17	0.564
BMI (kg/ m^2^)	25.8 ± 3.3	25.7 ± 3.4	25.9 ± 3.3	0.849
AF characteristics				
Paroxysmal AF, n (%)	67 (70.5%)	17 (65.4%)	50 (72.5%)	0.502
Persistent AF, n (%)	28 (29.5%)	9 (34.6%)	19 (27.5%)	0.502
CHA_2_DS_2_-VASc score ≥ 2	61 (64.2%)	16 (61.5%)	45 (65.2%)	0.740
HASBLED score ≥ 3	25 (26.3%)	8 (30.8%)	17 (24.6%)	0.547
First catheter ablation	85 (89.5%)	22 (84.6%)	63 (91.3%)	0.453
Medical history, n (%)				
Hypertension	60 (63.2%)	17 (65.4%)	43 (62.3%)	0.784
Diabetes mellitus	18 (18.9%)	8 (30.8%)	10 (14.5%)	0.073
History of ischemic heart disease	41 (45.5%)	10 (38.5%)	31 (44.9%)	0.573
Hyperlipidemia	70 (73.7%)	18 (69.2%)	52 (75.4%)	0.547
Stroke, TIA or thromboembolism	20 (21.5%)	6 (23.1%)	14 (20.3%)	0.782
Cigarette	33 (34.7%)	11 (42.3%)	22 (31.9%)	0.344
Alcohol	29 (30.5%)	11 (42.3%)	18 (26.1%)	0.128
Medications, n (%)				
Antiarrhythmic drugs	79 (83.2%)	19 (73.1%)	60 (87.0%)	0.109
Anticoagulation therapy	93 (97.9%)	26 (100%)	67 (97.1%)	0.383
Aspirin	26 (27.4%)	8 (30.8%)	18 (26.1%)	0.650
Calcium channel blockers	36 (37.9%)	11 (42.3%)	25 (36.2%)	0.588
ACEi/ARB	40 (42.1%)	14 (53.8%)	26 (37.7%)	0.157
Beta blocker	58 (61.1%)	17 (65.4%)	41 (59.4%)	0.597

*BSA* Body surface area, *BMI* Body mass index, *AF* Atrial fibrillation, *ACEi/ARB* Angiotensin-converting enzyme inhibitor/angiotensin receptor blocker

### Echocardiographic parameters

Some echocardiographic parameters were of significant difference between the 2 groups in AF patients with recurrence or without (Table 2). The recurrence group showed a larger LAVmax, LAVmin and LAVI than the no-recurrence group. Patients who experienced AF recurrence had lower LAEF, LASr, and LASct compared with those who did not (*P* < 0.05 for all). In addition, a similar appearance happened in paroxysmal AF patients. The recurrence group still presented a larger LAVmin and lower LAEF, LASr and LASct than the no-recurrence group in paroxysmal AF patients (Table 3). But it was not happened in persistent AF patients. However, both groups of AF patients, with or without recurrence, had comparable LV size and function.

**Table 2:** Echocardiographic characteristics of patients with AF recurrence versus those with AF no-recurrence

Variables	Total (*N* = 95)	Recurrence (*N* = 26)	No-Recurrence (*N* = 69)	*P* value
LAD	39.5 ± 5.1	40.9 ± 5.1	38.9 ± 5.0	0.093
LAVmax	58.8 ± 15.2	65.5 ± 16.9	56.3 ± 13.8	**0.008**
LAVmin	35.7 ± 14.3	43.4 ± 16.2	32.7 ± 12.5	**0.001**
LAVI	32.4 ± 7.9	35.5 ± 8.9	31.3 ± 7.2	**0.023**
LAEF	43.0 (29.0–51.0)	34.5 (21.0–45.8)	45.0 (33.5–52.5)	**0.013**
LASr	19.4 ± 8.3	16.6 ± 7.3	20.4 ± 8.4	**0.047**
LAScd	10.0 (8.0–14.0)	11.7 ± 3.7	10.0 (8.0–14.5)	0.317
LASct	9.0 (1.0–13.0)	2.5 (0.0–8.8)	11.0 (2.5–14.0)	**0.003**
E/e’	9.1 (7.2–11.8)	9.2 (7.2–11.3)	8.9 (7.2–11.8)	0.993
LVEDVI	37.6 ± 8.7	38.8 ± 11.1	37.2 ± 7.7	0.435
LVESVI	15.3 (13.6–18.3)	16.5 (14.4–18.3)	14.8 (13.2–18.3)	0.129
LVEF	58 (53–62)	56.0 (52.0–60.5)	60.0 (53.0–64.0)	0.053
LVGLS	17.8 (14.4–19.9)	16.9 (13.2–19.5)	18.0 (14.9–20.1)	0.499

*LA* Left atrium, *LAD* Left atrial diameter, *LAVmax* Maximal left atrial volume, *LAVmin* Minimal left atrial volume, *LAVI* Left atrial volume index, *LAEF* Left atrial ejection fraction, *LASr* LA reservoir strain, *LAScd* LA conduit strain, *LASct* LA contract strain, *LVEDVI* Left ventricular end diastolic volume index, *LVESVI* Left ventricular end systolic volume index, *LVEF* Left ventricular ejection fraction, *LVGLS* Left ventricular global longitudinal strain

**Table 3:** Relationship between LA structure and function with recurrence of AF patients after catheter ablation, stratified by AF subtype

AF subtype	variables	Recurrence	No-Recurrence	*P* value
PAF	LAVmax	57.0 (49.5–72.5)	52.0 (44.0–61.0)	0.069
	LAVmin	36.9 ± 15.3	27.4 ± 7.6	**0.001**
	LAVI	30.6 (26.7–42.0)	28.9 (24.9–34.1)	0.159
	LAEF	41.8 ± 12.3	48.4 ± 7.7	**0.012**
	LASr	20.2 ± 6.3	24.0 (21.0–28.0)	**0.016**
	LASct	7.3 ± 5.1	11.8 ± 4.9	**0.002**
perAF	LAVmax	71.5 ± 13.6	64.7 ± 14.5	0.243
	LAVmin	55.7 ± 9.6	46.7 ± 12.2	0.064
	LAVI	38.4 ± 6.8	35.9 ± 7.5	0.435
	LAEF	21.8 ± 6.8	27.9 ± 7.8	0.055
	LASr	9.9 ± 3.1	9.6 ± 2.7	0.789
	LASct	0.0 (0.0–0.5)	1.0 (0.0–4.0)	0.188

*LAVmax* Maximal left atrial volume, *LAVmin* Minimal left atrial volume, *LAVI* Left atrial volume index, *LAEF* Left atrial ejection fraction, *LASr* LA reservoir strain, *LAScd* LA conduit strain, *LASct* LA contract strain

### The prognostic value of echocardiographic parameters in AF recurrence

Univariate regression analyses were used to evaluate the association between AF recurrence and left cardiac volumetric and functional parameters, with significant correlative parameters included into a multivariable logistic regression analysis. Several conventional and strain parameters with odds ratio, 95% CI, and *P* values of prediction in AF recurrence were summarized in Table 4. Lower LASct was the only predictor of AF recurrence whether in the whole AF population or paroxysmal AF (*P* < 0.05), whereas enlarged LA volume, LAVI, reduced LA reservoir strain and LA conduit strain showed a prognostic trend toward AF recurrence but hadn’t reached statistical significance.

**Table 4:** Univariate and multi-variate logistic regression results for factors associated with AF recurrence

	Univariable (OR, 95% CI)	*P* value	Multivariable (OR, 95% CI)	*P* value
LAVmax	1.04 (1.01–1.07)	0.013		
LAVmin	1.05 (1.02–1.09)	0.002		
LAVI	1.07 (1.01–1.14)	0.029		
LAEF	0.95 (0.92–0.99)	0.011		
LASr	0.94 (0.89–1.00)	0.050		
LASct	0.89 (0.82–0.96)	0.004	0.89 (0.82–0.97)	0.007

*LAVmax* Maximal left atrial volume, *LAVmin* Minimal left atrial volume, *LAVI* Left atrial volume index, *LAEF* Left atrial ejection fraction, *LASr* LA reservoir strain, *LAScd* LA conduit strain, *LASct* LA contract strain

### Determination of cutoff value of LASct in AF recurrence

**Fig. 3:**
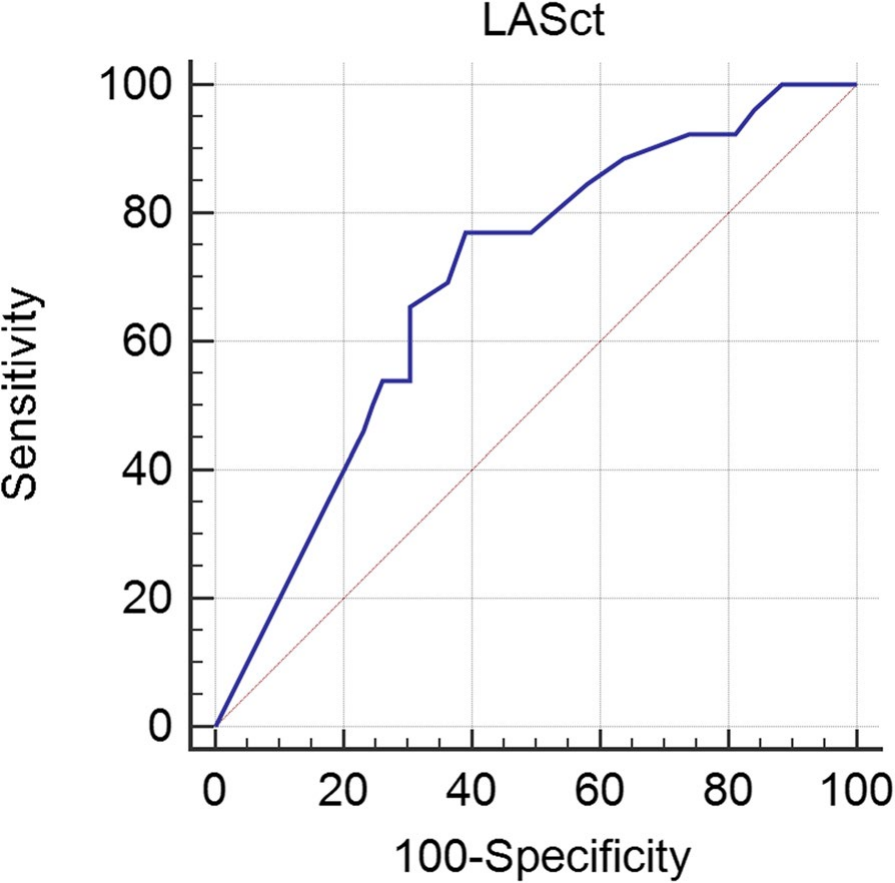
Receiver operating characteristic (ROC) analysis for the prognostic utility of LASct

Receiver operating characteristic analysis inhibited that LASct had a capability to identify the risk of recurrence in AF patients after catheter ablation, with an area under the curve of 0.70 (95% CI 0.59–0.79, *P* = 0.0008), sensitivity of 76.92%, and specificity of 60.87% (Fig. 3). The resulting cutoff value of LASct was 8%.

### Determination of reproducibility

**Fig. 4:**
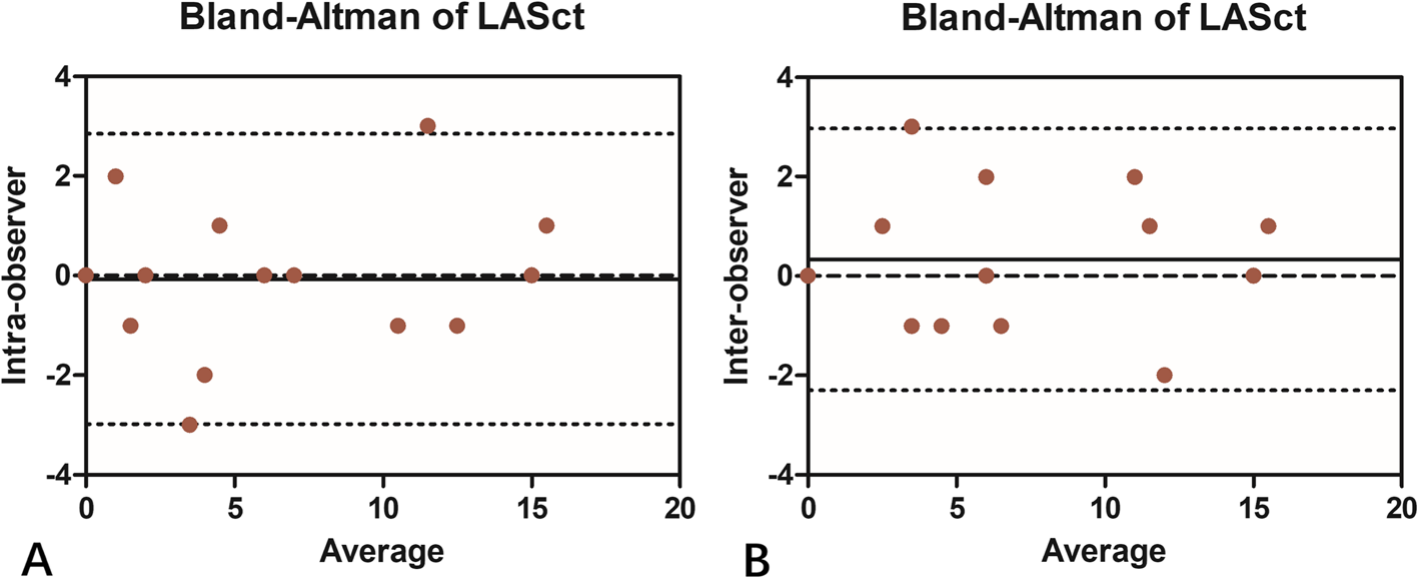
Bland-Altman plots depicting intra-observer (**A**) and inter-observer (**B**) variability of LASct

The intra- and inter-observer interclass correlation coefficients for LASct were 0.96 (0.89–0.98) and 0.96 (0.90–0.98), respectively. The mean intra- and inter-observer differences were presented by bias of − 0.06 ± 1.48 (95% CI, − 2.98-2.84) and 0.33 ± 1.34 (95% CI, − 2.30-2.96) for LASct, respectively (Fig. 4).

## Discussion

Our study indicates that LA strains assessed by STE can predict AF recurrence after CA. We observed that patients with a recurrence of non-valvular AF showed an increased LA volume and impaired LA function, and LASct demonstrated independently associated with recurrence after CA during follow-up. Further, non-valvular AF patients with a LASct < 8% exhibited a marked decrease in event-free survival for the recurrence at follow-up compared with patients remained in sinus rhythm. In addition, we did not find the independent prognostic value of LASct among persistent AF patients in the subgroup analysis of this study.

### Potential mechanisms of recurrence in patients with AF after catheter ablation

As catheter ablation is effective in restoring sinus rhythm and improving symptoms in therapy of AF, there still a probability of recurrence after the procedure in clinical practice. In our study, 27.4% of the cohort had a recurrence. That is consistent with prior studies, which showed similar recurrent rate after success of catheter ablation [15–18]. Potential mechanisms of recurrence may contain atrial fibrosis progression and electrical reconnection of isolated pulmonary veins due to previous catheter ablation, as well as ablation approach [19, 20]. Several predictors reported in previous studies for AF recurrence, such as age, CHA2DS2-VASc score, subtype of AF, LA size, and LA strain, were associated with atrial fibrosis and it was a step of atrial remodeling [21, 22]. Atrial fibrosis as quantified by LGE-MRI is common in AF patients and was demonstrated an independent risk factor for recurrence after catheter ablation at a long follow-up [23, 24]. During rapid atrial pacing at the episode of AF, some growth factors and peptides may promote the synthesis of collagen in atrial interstitium and alter the hemodynamics [25]. Masaya et al. [26] found that Ang II may promote the activation of MAPKs and expression of cathepsin K, leaving the aggradation of collagen in atrial fibrosis of AF. In their study, cathepsin K levels had a positive relation with left atrial diameter that measured by echocardiography, bringing about the alternation of atrial structure and reduced atrial compliance. LA strains measured by speckle tracking echocardiography were deemed as a surrogate of atrial fibrosis and remodeling, which may be available in the risk stratification of AF patients [27]. In addition, some individual risks, such as higher body mass index, thicker pericardial fat, obesity and obstructive sleep apnea would contribute to the recurrence of AF as well [19, 28].

### Risk assessment of recurrence in patients with non-valvular AF

Although increased LAVI and reduced LAEF in recurrence group was of statistical differences compared with no-recurrence group in our study and showed a predictive trend, they were not independent predictors. Previous studies have revealed that LASr had the ability in predicting AF recurrence after catheter ablation [3, 29, 30]. In another systematic review including 1025 patients with paroxysmal or persistent AF, Anne et al. [31] similarly showed that lower peak atrial longitudinal strain measured by 2D-STE was of independent association with high risk of recurrence following catheter ablation. In the present study, we investigated the LA function and found that LASct to have a good association with recurrence. In addition, LASct < 8% is independently associated with recurrence of AF patients underwent catheter ablation at follow-up. LASct is used for evaluation of LA contractile function and is affected by atrial contractility, preload and afterload [10]. As reduced or absent LA contractile function is frequent in AF patients, it was demonstrated that an LASct < 11.1% showed an increased incidence of recurrence after catheter ablation compared with those with an LASct ≥11.1% in a large cohort of 678 patients [32]. In the Copenhagen City Heart Study, the lower limits of normal LASct in 50–64 years old people were 9.2% for males and 6.8% for females [33]. The lower limit value is similar to our cutoff value of LASct. Meanwhile, a total of 2016 patients were selected in their secondary analysis to investigate the prognostic value of LA strain, with a 66-year of median age and 52% of female comparable to our study, shown that the lower limit of LASct was 13.2% in the prediction of AF during a period of 5.2 years at follow-up. Differences of statistical methods (95% confidence interval or cutoff value analyzed by receiver operating curve), individuals and heterogeneity of sample sizes may be account for the different cutoff values of LASct associated with recurrence of AF.

### Clinical implications

Our study indicates that LASct, evaluated by 2D-STE, may help to the risk stratification in AF patients. LA strains, especially LASct, should be applied to assess the status of AF patients whether they are suitable for a catheter ablation. Therefore, it is possible that LASct has the ability to identify the target patients who really need a timely effective catheter ablation to keep in a sinus rhythm. To some extent, this could avoid those ineffective procedures and save expenses. For the partial patients of not benefiting from catheter ablation, some other operational means should be considered and closer follow-up is also necessary. Moreover, in subgroups analysis of our study, the prognostic value of LASct was embodied in paroxysmal AF patients but not in persistent AF group. Due to a reduced or almost disappeared LA contractile function in persistent AF patients, the difference of LASct between two groups is of small significance, leading to unobvious predictive performance. This point should be taken into account to explain the results. In this regard, we ought to pay more attention on LA function of paroxysmal AF and choose the appropriate time to carry out a catheter ablation for patients. Furthermore, the inclusion of STE in conventional clinical practice would increase the detection rate of damaged LA function among AF patients and aid the therapeutic strategies.

### Limitations

Some inherent limitations include that this is a retrospective single-center small design. Under that, extra clinical information was not completely acquired. Thus, a prospective big multicenter cohort would be helpful for the accuracy of this results. Also, asymptomatic AF episodes or failure of capture by electrocardiogram during regular examination at follow-up may underestimate the recurrence rate. In addition, a two-year follow-up period is not enough and prolong the span may contribute to the occurrence of adverse events. On account of the retrospective nature of this study, we did not have additional information about laboratory indices and cardiac magnetic resonance data. Emphasis on LA function lead to neglect of the ablation details, and more details would be added to further study. Finally, thin LA walls are not easy to track, but the new dedicated LA-tracking software could improve the LA wall image contouring and reduce the intra- and interobserver variability.

## Conclusions

LASct was proved an independent predictor of AF recurrence after catheter ablation. The STE technique can be applied for risk stratification and selection of catheter ablation for AF patients.

## Data Availability

The datasets of this research are not publicly available due to the restrictions by the Beijing Chaoyang Hospital. The authors used this dataset under an agreement with the Beijing Chaoyang Hospital for the current study. The data are available from the corresponding author on reasonable request.

## Abbreviations

CA: Catheter ablation
AF: Atrial fibrillation
LA: Left atrium
STE: Speckle tracking cardiography
LASr: LA reservoir strain
LAScd: LA conduit strain
LASct: LA contractile strain
LAVmax: Maximum volume of LA
LAVmin: Minimum volume of LA
LAVI: LA volume index
LVEDVI: Left ventricular end-diastolic volume index
LVESVI: Left ventricular end-systolic volume index
LVEDV: Left ventricular end-diastolic volume
LVESV: Left ventricular end-systolic volume
BSA: Body surface area
LAEF: Left atrial ejection fraction
LAV: LA volume
ROC: Receiver-operator curves
